# Asociación entre preocupación frente a la COVID-19, el apoyo social y el conocimiento sobre tuberculosis con el cumplimiento del tratamiento antituberculoso en Lima, Perú

**DOI:** 10.7705/biomedica.6667

**Published:** 2023-06-30

**Authors:** Yuli L. Quiroz, Susan O. Choqueza, Anderson N. Soriano-Moreno, Jorge L. Alave

**Affiliations:** 1 Escuela de Medicina, Facultad Ciencias de la Salud, Universidad Peruana Unión, Lima, Perú Universidad Peruana Unión Escuela de Medicina, Facultad Ciencias de la Salud Universidad Peruana Unión Lima Peru; 2 Unidad de Investigación Clínica y Epidemiológica, Escuela de Medicina, Universidad Peruana Unión, Lima, Perú Universidad Peruana Unión Unidad de Investigación Clínica y Epidemiológica, Escuela de Medicina Universidad Peruana Unión Lima Peru

**Keywords:** COVID-19, tuberculosis pulmonar, cooperación del paciente, apoyo social, COVID-19, tuberculosis, pulmonary, patient compliance, social support

## Abstract

**Introducción.:**

En el contexto de la pandemia por la COVID-19 es escasa la información de factores asociados al cumplimiento del tratamiento antituberculoso en las zonas de alta prevalencia de tuberculosis.

**Objetivo.:**

Evaluar si existe asociación entre el apoyo social, la preocupación por el contagio de COVID-19 y el conocimiento de la tuberculosis, frente al incumplimiento del tratamiento antituberculoso.

**Materiales y métodos.:**

Se trata de un estudio transversal de pacientes en tratamiento antituberculoso durante los meses de enero a marzo del 2022 en centros ubicados en áreas de alta prevalencia de tuberculosis en Lima. Se utilizó el cuestionario de Morisky Green-Levine para evaluar el cumplimiento del tratamiento como variable dependiente; las variables independientes se evaluaron usando el *Medical Outcomes Study Social Support Survey* para determinar el apoyo social percibido y la preocupación por la infección de COVID-19, y el test de Batalla para evaluar el conocimiento del paciente sobre su enfermedad. Se utilizó la regresión de Poisson con varianza robusta para determinar la asociación entre las variables.

**Resultados.:**

De un total de 101 participantes (73,3 % hombres y edad media 35,1 ± 16 años), el 51,5 % no observaron el tratamiento antituberculoso. El nivel de preocupación medio o alto de contagiarse y desarrollar COVID-19 se asoció con una mayor prevalencia de incumplimiento del tratamiento (razón de prevalencia: 1,68; intervalo de confianza del 95 %: 1,09-2,57) (ajustada por las variables de confusión consideradas).

**Conclusiones.:**

El incumplimiento del tratamiento antituberculoso es una condición frecuente entre los pacientes de una zona de alta prevalencia de tuberculosis en Lima especialmente entre aquellos con mayor preocupación al contagio por el virus de SARS- CoV-2, causante de la COVID-19.

## Abstract (en)

**Introduction.:**

In the context of the COVID-19 pandemic, information on factors associated with adherence to antituberculosis treatment in areas with high prevalence of tuberculosis is scarce.

**Objective.:**

To evaluate whether there is an association between social support, concern about COVID-19 infection and knowledge about tuberculosis, and non-adherence to antituberculosis treatment.

**Materials and methods.:**

A cross-sectional study was carried out on patients under antituberculosis treatment, from January to March, 2022, in centers located in areas with a high prevalence of tuberculosis in Lima. We used the Morisky Green-Levine questionnaire to assess adherence to treatment as the dependent variable; the independent variables were evaluated using the Medical Outcomes Study Social Support Survey for perceived social support and concern about COVID-19 infection, and the Battle Test to assess patients’ knowledge about their disease. We used Poisson regression with robust variance to evaluate the association between the independent variables and the dependent one.

**Results.:**

Out of 101 participants (73.3% male with an average age of 35.1 ±16 years), 51.5% were non-adherent to antituberculosis treatment. Medium or high level of concern about getting COVID-19 was associated with a higher prevalence of non-adherence to treatment (odds ratio: 1.68; 95 % confidence interval: 1.09-2.57) (adjusted for considered confounding variables).

**Conclusions.:**

Non-adherence is a frequent condition among patients living in an area with a high prevalence of tuberculosis in Lima, especially among those with a higher concern for COVID-19 infection.

En el Perú, el Programa Nacional de Prevención y Control de Tuberculosis continúa implementando estrategias con la finalidad de controlar y reducir los casos nuevos de tuberculosis ^1^. La pandemia por la COVID-19 afectó los ámbitos social, económico y de salud pública ^2^. Se redujo la oferta de servicios de salud en el primer, el segundo y el tercer nivel de atención. Además, se restringieron las consultas y el seguimiento de los pacientes con enfermedades crónicas, limitando la atención a solo urgencias y emergencias relacionadas con estas enfermedades ^3^.

Para el Ministerio de Salud peruano, el abordaje de la COVID-19 fue prioridad, lo que provocó el debilitamiento de la gestión del Programa Nacional de Prevención y Control de Tuberculosis, reduciendo las actividades de detección y prevención de casos nuevos de tuberculosis, el acceso a los medicamentos y a los métodos diagnósticos oportunos ^1^. Por otro lado, las medidas de distanciamiento social y la cuarentena, entre otras disposiciones creadas con la finalidad de atenuar el contagio de la COVID-19, generaron miedo, preocupación e incertidumbre por el riesgo de contagio ^4^. Al comparar el año 2019 con el 2020 de pandemia por COVID-19 en Perú, el porcentaje de casos diagnosticados de tuberculosis disminuyó de 89,1 % (32.970/37.000) a 66,4 % (24.581/37.000); y el de abandonos al tratamiento de tuberculosis resistente aumentó de 6,9 % (237/3.422) a 7,8 % (215/2.744), respectivamente ^5^. Esto representó una amenaza al cumplimiento del tratamiento antituberculoso ^6^.

Distintos estudios han encontrado que el cumplimiento del tratamiento antituberculoso depende de factores como la influencia de la familia, la supervisión de su medicación mediante el tratamiento bajo observación directa, el apoyo de la comunidad, el apoyo del hogar, el apoyo espiritual, la carga financiera y el conocimiento de la enfermedad, entre otros ^7^^,^^8^. Los más propensos al incumplimiento del tratamiento fueron aquellos con escaso apoyo familiar ^7^.

Asimismo, en otro estudio determinaron que, para lograr la cura de la tuberculosis, debe existir un sistema de apoyo social estable basado en la familia, que se constituye en un factor imprescindible para acompañar al paciente durante la duración del tratamiento ^9^. Por otro lado, otra forma de valorar el cumplimiento del tratamiento es mediante el conocimiento que el paciente posee sobre su enfermedad, reportándose que a mayor conocimiento mayor cumplimiento ^10^. Del mismo modo, se ha encontrado que un nivel educativo alto y el conocimiento de la tuberculosis tuvieron una asociación significativa con la observancia de la terapia antituberculosa ^11^.

El conocer los factores asociados con el cumplimiento del tratamiento antituberculoso en el contexto de la actual pandemia de COVID-19 constituye una información importante y necesaria debido al contexto social, económico y de salud pública. El factor de apoyo social desde el número de personas que conforman su entorno, las interacciones afectivo-emocionales y las actividades cotidianas que las conforman, son de gran interés para que el paciente cumpla su tratamiento, así como también, la preocupación por la posibilidad de contagiarse con el virus que genera la COVID-19.

Se llegaron a implementar estrategias de prevención y promoción en la lucha contra la pandemia como el distanciamiento social, el uso de mascarillas, el lavado de manos, la telemedicina en el primer nivel de atención, etc. Este contexto nos ha demostrado deficiencias en el sector salud que sirven como lecciones que deben ser analizadas y aprendidas con la finalidad de comprometernos a resguardar y diseñar futuras estrategias que mejoren la observancia del tratamiento en esta población.

Por las razones expuestas, este estudio tuvo el objetivo de evaluar la frecuencia del incumplimiento del tratamiento para la tuberculosis y explorar su asociación con el apoyo social, el conocimiento de la enfermedad y la preocupación por el contagio el virus que genera la COVID-19, en dos centros de primer nivel de atención y un hospital público de áreas de alta prevalencia de tuberculosis de Lima, Perú.

## Materiales y métodos

### Estudio, lugar y participantes

Se trata de un estudio transversal realizado de enero a marzo del 2022. Se encuestaron los pacientes pertenecientes al Programa Nacional de Prevención y Control de Tuberculosis de los centros de salud San Hilarión, San Cosme y del Hospital de Huaycán, ubicados en tres distritos de Lima: San Juan de Lurigancho, La Victoria y Ate-Vitarte, respectivamente.

En el 2019, Lima registró el 57,47 % (23.580/41.032) de los casos de tuberculosis del Perú y los establecimientos de salud en los que se realizó nuestra investigación se encuentran en los distritos de Lima con alta incidencia de tuberculosis ^12^. Por ejemplo, la jurisdicción del centro de salud San Cosme reporta altos niveles de hacinamiento y posee una tasa de morbilidad por tuberculosis de 1.347 por 100.000 habitantes, aproximadamente diez veces el promedio nacional ^13^.

Se incluyeron pacientes adultos de 18 a 70 años que recibieron tratamiento para tuberculosis sensible, multirresistente (MDR-TB) y extremadamente resistente (XDR-TB), al menos, por un mes. Se excluyeron los pacientes con tuberculosis extrapulmonar por la dificultad de su manejo sobre todo en centros de primer nivel de atención.

La muestra final estuvo compuesta por 101 pacientes, lo que nos permitió alcanzar una precisión del 10 % en la detección de una prevalencia esperada del 50 %, considerando un nivel de confianza del 95 % y asumiendo una población infinita. La selección de los pacientes se llevó a cabo mediante un método por conveniencia.

### Variable dependiente

Se consideró como variable dependiente el cumplimiento del tratamiento y para su análisis se utilizó el cuestionario de Morisky-Green-Levine, empleado para evaluar la observancia del tratamiento de distintas enfermedades como la hipertensión arterial, la del virus de inmunodeficiencia humana y la tuberculosis ^14^^-^^16^. Este cuestionario fue validado en su versión en español por Val Jiménez *et al.*^17^. Consta de cuatro preguntas con respuestas de no o sí. Este cuestionario define como cumplidor si el participante responde las cuatro preguntas con la siguiente secuencia de respuestas: no, sí, no, no. En caso contrario, se considera que no cumple.

### Variables independientes

Se consideraron como variables independientes el apoyo social, la preocupación de contagio por el virus causante de la COVID-19 y el conocimiento del paciente sobre la tuberculosis.

Para explorar el apoyo social se utilizó el cuestionario *Medical Outcomes Study Social Support Survey* (MOS-SSS), empleado con pacientes con enfermedades crónicas ^18^. La validación en español se hizo en diferentes países de Iberoamérica ^19^^,^^20^. Se compone de 20 ítems: la primera pregunta estima el apoyo social cuantitativo (número de personas que conforman su entorno social) y para este estudio usamos los 19 ítems posteriores. Estos valoran cualitativamente el apoyo social dividido en cuatro dimensiones: afectiva, interacción social positiva, instrumental y apoyo emocional. Esta herramienta categoriza los valores en apoyo social máximo (71 a 95 puntos), medio (45 a 70 puntos) y mínimo (19 a 44 puntos) ^21^.

Además, se utilizó la escala de preocupación por el contagio con el virus SARS-CoV-2, causante de la COVID-19 para evaluar su efecto en el estado anímico y en el desarrollo de las actividades cotidianas de los pacientes. Esta escala fue diseñada y validada por Caycho R. *et al.,* en Perú en 2020 ^22^. Este es un cuestionario que consta de seis preguntas con cuatro opciones de respuesta según una escala de calificación (tipo Likert). Para este estudio se categorizaron los resultados de este cuestionario en terciles: tercil uno o preocupación baja (6 a 11 puntos), tercil dos o preocupación media (12 a 14 puntos), tercil tres o preocupación alta (15 a 24 puntos).

Finalmente, se utilizó el cuestionario de Batalla para evaluar el conocimiento que tiene el paciente sobre su enfermedad. Este ha sido utilizado en enfermedades crónicas ^11^^,^^23^^,^^24^. Consta de tres preguntas y si responde correctamente la secuencia: no, sí, sí, se considera que el paciente conoce su enfermedad; si falla una de ellas, se considerará no conocedor ^25^.

### Covariables

También evaluamos las siguientes variables sociodemográficas: edad en años, sexo (masculino, femenino), nivel de estudios (secundaria completa, preuniversitario, universitario), comorbilidades (diabetes mellitus de tipo 2, VIH, hipertensión arterial, obesidad, hipotiroidismo, artritis reumatoidea), ocupación (estudiante, ama de casa, con empleo, pensionado o desempleado), estado civil (casado, soltero, viudo, divorciado, unión libre), institución (centros de salud San Hilarión, San Cosme y Hospital Huaycán) y tipo de esquema de tratamiento contra la tuberculosis (tuberculosis sensible y multirresistente o extremadamente resistente).

### Procedimiento de recolección de la información

Se coordinó con los directores de los establecimientos de salud para que los investigadores obtuvieran el consentimiento informado, aplicaran los cuestionarios y recolectaran la información demográfica y clínica de las fichas individualizadas de los pacientes que aceptaron participar en el estudio y que pertenecían al Programa Nacional de Prevención y Control de la Tuberculosis.

Los potenciales participantes fueron captados durante su asistencia al Programa Nacional de Control y Prevención de la Tuberculosis de los tres establecimientos de salud. Luego de autorizar el consentimiento informado se aplicaron los cuestionarios mencionados entrevistando a los participantes en cada centro de salud, luego de que recibieran su medicación antituberculosa.

### Análisis estadístico

Se describieron las variables categóricas con frecuencias absolutas y relativas, y las variables numéricas con media y desviación estándar. Se utilizó la prueba de ji al cuadrado en el análisis bivariado para comparar la proporción del incumplimiento del tratamiento según las categorías de las variables independientes. Además, se crearon modelos de regresión de Poisson con varianza sólida para evaluar la asociación entre el apoyo social, la preocupación por la COVID-19 y el conocimiento de la tuberculosis con la variable dependiente “incumplimiento del tratamiento”.

Se calcularon razones de prevalencias (RP) con sus intervalos de confianza del 95 % (IC_95%_) crudos y ajustados por sexo, edad, estado civil soltero, nivel de educación secundaria y presencia de comorbilidades, dado que estas variables se consideraron potencialmente de confusión con base en el criterio epidemiológico.

Se hizo un análisis estratificado según las categorías de las variables “apoyo social” y “conocimiento de la enfermedad tuberculosa”, considerando que podrían ser modificadoras del efecto de la preocupación frente a la COVID-19 y el incumplimiento. Se consideró un valor de p menor de 0,05 como significativo. Los análisis se realizaron con el programa estadístico R, versión 4.

### Aspectos éticos

El estudio fue evaluado y aprobado por el Comité de Ética Institucional de la Universidad Peruana Unión (código: 2021-CE-FCS-UPeU-00333). La encuesta fue anónima y voluntaria para los participantes previa firma del consentimiento informado.

## Resultados

### Características generales

De los 101 participantes, el 51,5 % no cumplió con el tratamiento. El sexo masculino predominó en el 73,3 % de los pacientes y la edad promedio fue de 35,1 ± 16 años. El 53,5 % tenía estudios secundarios completos, el 51,5 % eran solteros y el 22,8 % reportó alguna comorbilidad, la más frecuente fue la diabetes en el 11,9 %. Además, el 56,4 % resultó con apoyo social máximo, el 58,4 % era conocedor de su enfermedad y el 55,4 % tuvo un nivel de preocupación medioalto de contagiarse con el virus causante de la COVID-19 (cuadro 1).

**Cuadro 1:** Características demográficas de los participantes

Características		%(n)
	Sexo femenino	74 (73,3)
	Edad, media (desviación estándar)	35,1 (16,0)
	Secundaria completa	54 (53,5)
	Soltero	52 (51,5)
	Con comorbilidades	23 (22,8)
Institución		
	San Cosme	50 (49,5)
	San Hilarión	22 (21,8)
	Hospital Huaycán	29 (28,7)
Esquema		
	Sensible	81 (80,2)
	MDRoXDR	20 (19,8)
Ocupación		
	Estudiante	18 (17,8)
	Ama de casa o con empleo	55 (54,5)
	Pensionado o desempleado	28 (27,7)
Apoyo social (MOS-SSS)		
	Máximo	57 (56,4)
	Medio	37 (36,6)
	Mínimo	7 (6,9)
Cumple con el tratamiento (Morisky-Green)		
	No	52 (51,5)
	Sí	49 (48,5)
Conocedor de su enfermedad (Batalla)		
	No	42 (41,6)
	Sí	59 (58,4)
Preocupación frente a la COVID-19		
	Baja (6 a 11 puntos)	45 (44,6)
	Media (12 a 14 puntos)	28 (27,7)
	Alta (15 a 24 puntos)	28 (27,7)

### Análisis bivariado

Los participantes que tuvieron una preocupación media o alta por la COVID-19 observaron el tratamiento en el 33,6 % y un 15,8 % menos que aquellos con una preocupación baja (p=0,019). Por otro lado, los participantes que tenían un apoyo social medio o mínimo mostraban menos cumplimiento del tratamiento en el 13,9 y el 11,5 % respectivamente, en comparación con aquellos que tenían un apoyo social máximo (p=0,379). Y de aquellos participantes que no conocían su enfermedad, cumplen con el tratamiento un 13,7 % menos respecto a aquellos participantes que conocían su enfermedad (cuadro 2).

**Cuadro 2:** Apoyo social, preocupación por la infección de COVID-19 y conocimiento sobre la tuberculosis, según el cumplimiento del tratamiento

Variables		Cumplimiento del tratamiento (Morisky-Green)		p
		Sí (N=49) n (%)	No (N=52) n (%)	
Apoyo social (MOS-SSS)				0,379
	Máximo	31 (54,4)	26 (45,6)	
	Medio	15 (40,5)	22 (59,5)	
	Mínimo	3 (42,9)	4(57,1)	
Preocupación frente a la COVID-19				0,019
	Baja	28 (62,2)	17 (37,8)	
	Media	8 (28,6)	20 (71,4)	
	Alta	13 (46,4)	15 (53,6)	
Conocedor de su enfermedad (Batalla)				02,45
	Sí	32 (54,2)	27 (45,8)	
	No	17 (40,5)	25 (59,5)	

* Valor de p calculado con la prueba de ji al cuadrado

### Análisis multivariado

En el análisis de regresión de Poisson observamos que aquellos que tenían una preocupación media o alta por la COVID-19 presentaron una prevalencia de incumplimiento del tratamiento 1,91 veces (IC_95%_: 1,222,98) y 1,42 veces (IC_95%_: 0,852,36) más comparados con aquellos que tenían una baja preocupación. Al combinar las categorías media y alta encontramos que estos participantes veces menos al tratamiento (IC_95%_: 1,25-6,667) comparado con los de la categoría de preocupación baja. Además, se observó que los participantes que tenían un apoyo social medio (RP=1,35; IC_95%_: 0,902,04) o mínimo (RP=1,27; IC_95%_: 0,65-2,47) cumplieron menos con el tratamiento antituberculoso en comparación con aquellos que tenían un apoyo social máximo; aquellos que no conocían su enfermedad tuvieron una prevalencia de 1,32 veces menor (IC_95%_: 0,89-1,95) de cumplimiento en relación con aquellos que sí conocían de su enfermedad. Sin embargo, ninguno de estos resultados fue estadísticamente significativo (cuadro 3).

**Cuadro 3:** Análisis multivariado entre el incumplimiento del tratamiento antituberculoso, el conocimiento de la tuberculosis, el apoyo social y la preocupación por la COVID-19

		Modelos crudos			Modelos ajustados*		
Factores		^RR^	^IC^_95%_	^p^	^RR^	^IC^_95%_	^p^
Apoyo social (MOS-SSS)							
	Máximo	1,00			1,00		
	Medio	1,30	0,88 - 1,92	0,181	1,35	0,90 - 2,04	0,150
	Mínimo	1,25	0,62 - 2,53	0,529	1,27	0,65 - 2,47	0,487
Preocupación frente a la COVID-19							
	Baja	1,00			1,00		
	Media	1,89	1,22-2,94	0,005	1,91	1,22-2,98	0,004
	Alta	1,42	0,85 - 2,36	0,179	1,42	0,85 - 2,36	0,181
Preocupación frente la COVID-19							
	Baja	1,00			1,00		
	Media-alta	1,65	1,08 - 2,53	0,021	1,68	1,09 - 2,57	0,018
Conocedor de su enfermedad (Batalla)							
	Sí	1,00			1,00		
	No	1,30	0,90-1,89	0,167	1,32	0,89-1,95	0,165

* Modelos ajustados por edad en años, sexo, educación secundaria, estado civil y presencia de comorbilidades.

### Análisis de modificación de efecto

Se observó que la asociación entre la preocupación frente a la COVID-19 y el incumplimiento se mantuvo estadísticamente significativa entre aquellos con un apoyo social medio (RP=2,42; IC_95%_: 1,14-5,15), pero perdió significancia estadística entre aquellos con un apoyo social máximo (RP=1,63; IC_95%_: 0,85-3,10). Al estratificar entre aquellos con conocimientos de la enfermedad tuberculosa y sin ella, se observó que la asociación entre preocupación por COVID-19 e incumplimiento incrementó su significancia estadística entre aquellos que no tenían conocimientos sobre la enfermedad (RP=2,12; IC_95%_: 1,15-3,93), más no entre aquellos con conocimientos (RP=1,37; IC_95%_: 0,76-2,44) (cuadro 4).

**Cuadro 4:** Evaluación de la asociación entre la preocupación frente a la COVID 19 y el incumplimiento del tratamiento antituberculoso, según apoyo social y conocimiento de la enfermedad

		Modelos crudos			Modelos ajustados*		
Factores		^RR^	^IC^_95%_	^p^	^RR^	^IC^_95%_	^p^
Apoyo social máximo							
	Preocupación frente a la COVID-19						
	Baja	1,00			1,00		
	Media o alta	1,70	0,92-3,16	0,093	1,63	0,85-3,10	0,139
Apoyo social medio							
	Preocupación frente a la COVID-19						
	Baja	1,00			1,00		
	Media o alta	2,16	0,94-4,99	0,071	2,42	1,14-5,15	0,022
Apoyo social mínimo								
	Preocupación frente a la COVID-19							
	Baja	1,00			1,00			
	Media o alta	-			-			
Conoce de su enfermedad								
	Preocupación frente la COVID-19							
	Baja	1,00			1,00			
	Media o alta	1,34	0,74-2,41	0,330	1,37	0,76-2,44	0,293	
No conoce su enfermedad							
	Preocupación frente la COVID-19						
	Baja	1,00			1,00		
	Media o alta	2,12	1,13-3,98	0,018	2,12	1,15-3,93	0,016

* Modelos ajustados por edad en años, sexo, educación secundaria, estado civil y presencia de comorbilidades.

## Discusión

Evaluamos si durante la pandemia por COVID-19, el apoyo social, la preocupación a contagiarse con el virus causante de la COVID-19 y el conocimiento de tuberculosis estaban asociados al incumplimiento del tratamiento antituberculoso en zonas de alta prevalencia de la enfermedad en Lima. Se encontró que más de la mitad de los encuestados no observaron el tratamiento antituberculoso, especialmente aquellos con nivel medio-alto de preocupación de contagio. Por otra parte, observamos que esta asociación no tenía relevancia significativa en aquellos con un apoyo social máximo y entre aquellos que conocían sobre la enfermedad tuberculosa.

Con respecto a la frecuencia alta de participantes que incumplieron con el tratamiento antituberculoso encontrada en nuestro estudio, Velásquez *et al.* describieron también un alto porcentaje de pacientes con tuberculosis que lo habían hecho (67,3 %) en Perú. Utilizaron el cuestionario de Morisky- Green-Levine y realizaron el estudio durante la pandemia de COVID-19 ^26^. Contrario a lo reportado en tiempos previos a esta pandemia, el estudio europeo de Khomova *et al.* aplicó el cuestionario de Morisky-Green-Levine en pacientes con TBC-MDR y encontraron que el 55 % habían cumplido con el tratamiento ^27^. Asimismo, en África, Fagundez *et al.* determinaron que el cumplimiento del tratamiento antituberculoso había sido de 78,57 % ^11^.

La variación de los niveles de cumplimiento entre los diversos estudios, puede explicarse por el contexto de la pandemia de COVID-19 que provocó serias dificultades en los sistemas de salud de los países con alta prevalencia de tuberculosis y en nuestro estudio particularmente debido a la preocupación de contagio por el virus causante de la COVID-19. No obstante, en Argentina, De Pereira *et al.* reportaron una experiencia interesante para mejorar el cumplimiento del tratamiento mediante el seguimiento activo vía telefónica durante el 2020 y 2021, demostrando que, a pesar de las medidas de aislamiento social preventivo y obligatorio, se pudo alcanzar un porcentaje de cumplimiento del tratamiento antituberculoso del 70 %, aproximadamente ^28^.

Se requiere más información de la tasa de observancia del tratamiento antituberculoso y del reporte de estrategias que hayan permitido mantener niveles altos de cumplimiento en las zonas de alta prevalencia de tuberculosis en el contexto de la pandemia de COVID-19.

Un hallazgo novedoso de nuestro estudio fue la preocupación de contagiarse con el agente etiológico de COVID-19 como factor asociado al incumplimiento del tratamiento antituberculoso. Este factor ha sido evaluado en otros contextos; por ejemplo, en los estudios realizados con enfermeras, Carranza *et al.* encontraron que la ansiedad y la preocupación de contagio estaban asociados a la depresión ^29^. Otro estudio de Carranza y colaboradores, en el cual se buscaba validar la escala de preocupación por contagio del virus SARS-CoV-2, encontró que al salir del trabajo, por el personal de salud se preocupaba por contagiar a familiares o personas con las que convivían ^30^^,^^31^.

Hasta donde sabemos, este es el primer estudio que destaca a la preocupación de contagio del coronavirus causante de la COVID-19 como factor asociado al cumplimiento del tratamiento antituberculoso. Esto se puede explicar por la desinformación y el miedo producido por los medios de comunicación que puede llevar a sobreestimar el riesgo de contagio. Por esta razón, los pacientes con tuberculosis pueden temer exponerse a personas que pudieran tener COVID-19 y, por lo tanto, continúan en aislamiento ^32^. En nuestro estudio, los pacientes con preocupación de contagio por SARS- CoV-2 no acudieron regularmente a recibir sus medicamentos o abandonaron su tratamiento.

La asociación entre la preocupación frente a la COVID-19 y el incumplimiento del tratamiento no se observó en aquellos con un apoyo social máximo o aquellos que conocían la enfermedad tuberculosa. Esto es similar a un estudio publicado en Colombia sobre los factores relacionados con el cumplimiento del tratamiento antituberculoso. Dueñes señala que la carga social es un factor subjetivo relacionado al cumplimiento del tratamiento mientras los factores objetivos son la afiliación con el sistema de salud y la tolerancia a los medicamentos ^33^.

El apoyo social es un soporte para que los pacientes puedan combatir la tuberculosis ^34^, ya que se asoció con buenos niveles de cumplimiento del tratamiento en enfermedades crónicas como la infección por VIH, las enfermedades neoplásicas, la hipertensión arterial, la diabetes mellitus y la tuberculosis ^35^^-^^38^.

Chen Xu *et al.* evaluaron cumplimiento del tratamiento contra la tuberculosis en pacientes con reciente diagnóstico y encontraron que una mejor observancia estaba asociada con tener familiares que supervisaran la medicación, brindaran aliento espiritual, tuvieran conocimiento relacionado con la tuberculosis. Otro factor clave fue el apoyo de las políticas del Estado para el control de la tuberculosis ^39^.

Por otro lado, el desconocimiento de la enfermedad tuberculosa fue otra característica entre los participantes con preocupación frente a la COVID-19. Algunos estudios mencionan que el tener un mayor conocimiento de la tuberculosis, como por ejemplo conocer que la tuberculosis es curable, fomenta el deseo de curarse y, por ende, de completar el tratamiento ^40^. De igual forma, el conocimiento de diferentes enfermedades crónicas por parte de los pacientes contribuyó positivamente con el cumplimiento del tratamiento de estas ^41^^,^^42^.

El hallazgo de la interacción de la preocupación de contagio con el apoyo social y el nivel de conocimiento de la enfermedad tuberculosa sugiere que las estrategias deben continuar reforzando las políticas de apoyo social y familiar, así como programas de capacitación de conocimiento de la enfermedad tanto para los pacientes y la comunidad en zonas de alta endemia de tuberculosis. Se requieren estudios con mayor tamaño de muestra con la finalidad de verificar nuestros hallazgos y encontrar otras potenciales asociaciones e interacciones.

Nuestro estudio tuvo las siguientes limitaciones: primero, el tamaño de la muestra fue pequeño y se seleccionó por conveniencia, lo que limita la extrapolación de los resultados a otras poblaciones y produce el riesgo de error tipo dos. Segundo, dado que los datos fueron referidos por los participantes, existe la posibilidad de sesgo en la información. No se revisaron las historias clínicas, ni las tarjetas de control del tratamiento antituberculoso, pero hubiese sido ideal. Sin embargo, los instrumentos utilizados han sido validados previamente y nos permiten explorar las variables propuestas. Tercero, aunque en el modelo multivariado incluimos algunas variables potencialmente de confusión, faltó ajustar los datos con el estado socioeconómico ya que esto podría afectar los resultados, por lo cual se recomienda incluir esta variable en el análisis de estudios futuros.

Por otra parte, nuestro estudio, cuenta con la fortaleza de ser uno de los primeros en analizar factores poco evaluados en investigaciones previas y de desarrollarse en una zona de alta prevalencia de tuberculosis.

En conclusión, en diversos estudios se ha descrito que existen factores determinantes relacionados al incumplimiento del tratamiento antituberculoso. Sin embargo, estos no se habían evaluado en el contexto de una pandemia que constituyó un problema de salud pública. Nuestros hallazgos sugieren que incumplimiento del tratamiento de la tuberculosis durante la pandemia fue frecuente y que el temor o preocupación frente a la COVID-19 podría ser un factor clave en estas circunstancia.

Por otra parte, un adecuado apoyo social y el conocimiento de la enfermedad por parte del paciente podrían ser factores que contribuyan a disminuir dicha asociación aumentando el cumplimiento del tratamiento. Aunque, el tamaño de la muestra de nuestro estudio fue pequeño para poder precisar asociaciones, debemos fomentar e impulsar la importancia de fortalecer el vínculo del apoyo social, la aplicación de programas de difusión de conceptos básicos sobre la tuberculosis, su cura y su tratamiento para un mejor cumplimiento de este.

## References

[1] Ministerio de Salud Peruano Dirección de Prevención y Control de Tuberculosis..

[2] Castro-Baca AM, Villena-Pacheco AE. (2021). La pandemia del COVID-19 y su repercusión en la salud pública en Perú. Acta Médica Peruana.

[3] Ministerio de Salud Peruano Resolución Ministerial N° 095-2020-MINSA RM_095- 2020-MINSA. Plan nacional de reforzamiento de los servicios de salud y contención del COVID-19.

[4] Quezada-Scholz VE. (2020). Miedo y psicopatología: la amenaza que oculta el Covid-19.. CNPs.

[5] Ríos-Vidal JRM. Situación actual- Estrategias de Prevención y Control de TBC en el Perú.

[6] Cárdenas-Escalante J, Fernández-Saucedo J, Cubas WS. (2022). Impacto de la pandemia por COVID-19 en la tuberculosis en el Perú: ¿nos estamos olvidando de alguien?. Enferm Infecc Microbiol Clin.

[7] Munro SA, Lewin SA, Smith HJ, Engel ME, Fretheim A, Volmink J. (2007). Patient adherence to tuberculosis treatment: a systematic review of qualitative research. PLoS Med.

[8] Bank AL, Indarwati R, Sulistiawati S. (2020). The role of social support on treatment adherence In Tb patients: a systematic review.. Nurse and Health: Jurnal Keperawatan.

[9] Saqib SE, Ahmad MM, Panezai S. (2019). Care and social support from family and community In patients with pulmonary tuberculosis In Pakistan. Fam Med Community Health.

[10] Martinez-Fajardo EJ, Garcia-Valdez R, Álvarez-Villaseñor AS. (2019). Adherencia al tratamiento farmacológico en pacientes con hipertensión arterial de un consultorio auxiliar. Med Gen Fam.

[11] Fagundez G, Perez-Freixo H, Eyene J, Momo JC, Biyé L, Esono T (2016). Treatment adherence of tuberculosis patients attending two reference units in Equatorial Guinea. PLoS One.

[12] Ministerio de Salud Peruano (2019). Vigilancia epidemiológica de tuberculosis.

[13] Fuentes-Tafur LA. (2009). Enfoque sociopolítico para el control de la tuberculosis en el Perú. Rev Peru Med Exp Salud Pública.

[14] García GM de M, Sit MS, Perera AE. (2020). Adherencia farmacológica en pacientes hipertensos. Rev Cubana Med Gen Integr.

[15] Suárez-Villa M, Lastre-Amell G, Rodríguez-López J. (2018). Adherencia a fármaco-terapia antirretroviral para el tratamiento del VIH/SIDA en la costa caribe colombiana. Revista Latinoamericana de Hipertensión.

[16] Meza-Condenzo W, Peralta-Pumapillo A, Quispe-Gómez F, Cáceres-Bellido FE. (2018). Adherencia terapéutica y factores condicionantes en su cumplimiento en pacientes con tuberculosis pulmonar atendidos en la Microred la Palma, lea 2017. Rev Méd Panacea.

[17] Nogués-Solán X, Sorli-Redó ML, Villar-García J. (2007). Instrumentos de medida de adherencia al tratamiento.. An Med Interna.

[18] Martín-Carbonell M, Cerquera-Córdoba A, Fernández-Daza M, Higuita JD, Galván- Patrignani G, Guerrero-Martel M (2019). Estructura factorial del Cuestionario de Apoyo Social MOS en ancianos colombianos con dolor crónico. Ter Psicol.

[19] Londoño-Arredondo NH. (2012). Validation of the Colombian MOS social support survey. Int J Psychol Res.

[20] Baca RD. (2016). Confiabilidad y validez del cuestionario de apoyo social en pacientes con cáncer de Trujillo.. Revista de Investigación en Psicología.

[21] Revilla Ahumada., Bailón- Muñoz E. El cuestionario Medical Outcomes Study (MOS), un instrumento para evaluar el apoyo social.

[22] Caycho-Rodríguez T, Ventura-León J, Barboza-Palomino M. (2021). Diseño y validación de una escala para medir la preocupación por el contagio de la COVID-19 (PRE-COVID-19). Enfermería Clínica.

[23] Vargas-Cárdenas G, Balvin-Yanes L, Chaiña-Meza JM, LlanosTejada F, Vargas- Cárdenas G, Balvin-Yanes L (2020). Adherencia terapéutica al tratamiento de erradicación de Helicobacter pylori y sus factores asociados en un hospital público de Perú. Rev Gastroenterol Peru.

[24] Aid Kunert J. (2015). Adherencia al tratamiento antihipertensivo en pacientes ambulatorios de un hospital urbano. Rev Virtual Soc Parag Med int.

[B25] Rodríguez Chamorro MÁ, García-Jiménez E, Amadles P, Rodríguez Chamorro A, José Faus M. (2008). Revisión de tests de medición del cumplimiento terapéutico utilizados en la práctica clínica. Aten Primaria.

[26] Velasquez-Yupanqui ID. (2021). Relación entre riesgo familiar y adherencia al tratamiento en pacientes con tuberculosis atendidos en el C.S. San Francisco de la Red de Salud de Tacna, en el marco de la pandemia de la COVID - 19 en el año 2020 (tesis).

[27] Khomova N, Tashpulatova F, Sultanov S. (2017). Compliance - is patient adherence to treatment, as well as partnerships between doctor and patient. Eur Respir J.

[28] Pereira AM. (2021). Seguimiento activo de casos de tuberculosis durante la pandemia de covid-19 en un hospital general de agudos de la ciudad de Buenos Aires. Revista Argentina de Medicina.

[29] Carranza-Esteban RF, Mamani-Benito O, Quinteros-Zúñiga D, Caycho-Rodríguez T, Blanco- Shocosh D. (2021). Preocupación por el contagio de la COVID-19, apoyo social en el trabajo y ansiedad como predictores de la depresión en enfermeras peruanas. Salud Uninorte.

[30] Carranza-Esteban RF, Mamani-Benito OJ, Corrales-Reyes IE, Landa-Barzola M, Marca-Dueñas G, Tito-Betancur VS (2021). Evidencias psicométricas de una escala de preocupación por el contagio de la COVID-19 en internos peruanos. Rev Cuba de Investig Biomed.

[31] Esteban-Carranza RF, Mamani-Benito OJ, Rodríguez-Alarcon JF, Corrales-Reyes IE, Farfán-Solís R. (2023). Escala de preocupación por el contagio de la COVID-19 en personal de la salud peruano. Rev Colomb Psiquiatr.

[32] Mejia CR, Ticona D, Rodríguez-Alarcon JF, Campos-Urbina AM, Catay-Medina JB, Porta-Quinto T (2020). The media and their informative role in the face of the coronavirus disease 2019 (COVID-19): Validation of fear perception and magnitude of the issue (MED- COVID-19). Electron J Gen Med.

[33] Dueñes M, Cardona D. (2016). Factors related to treatment adherence in patients with tuberculosis in Pereira, Colombia, 2012-2013. Biomédica.

[34] Potter JL, Inamdar L, Okereke E, Collinson S, Dukes R, Mandelbaum M. (2016). Support of vulnerable patients throughout TB treatment in the UK. J Public Health (Oxf).

[35] Huynh AK, Kinsler JJ, Cunningham WE, Sayles JN. (2013). The role of mental health in mediating the relationship between social support and optimal ART adherence. AIDS Care.

[B36] Coiiacso-Fiesta HR, Leon-Lamilla LL. (2018). Adaptación del Cuestionario MOS de Apoyo Social en pacientes oncológicos del Instituto Nacional de Enfermedades Neoplásicas (tesis).

[37] Oshiro Bernuy JH. (2019). Apoyo social percibido en pacientes con hipertensión arterial y diabetes mellitus tipo 2 en atención primaria y su relación con la adherencia al tratamiento (tesis).

[38] Solorzano LMS. (2019). Apoyo social en el tratamiento de la tuberculosis, Hospital Luis Uría de la Oliva, Caja Nacional de Salud, Gestión 2016 (tesis).

[39] Chen X, Du L, Wu R, Xu J, Ji H, Zhang Y (2020). The effects of family, society and national policy support on treatment adherence among newly diagnosed tuberculosis patients: a cross-sectional study. BMC Infect Dis.

[40] Wares DF, Singh S, Acharya AK, Dangi R. (2003). Non-adherence to tuberculosis treatment in the eastern Tarai of Nepal. Inf J Tubero Lung Dis.

[41] Despaigne RM, Varona DTC. (2018). Adherencia terapéutica en pacientes con enfermedades crónicas hospitalizados en un servicio de Medicina Interna. J Pharm Pharmacogn Res.

[42] Moreano Molina AP. (2019). Test de Morisky-Green-Levine y Batalla en diabéticos del Hospital Rafael Ruiz de Pujilí y propuesta educativa (tesis ].

